# Identification and Validation of Ferroptosis-Related Biomarkers in Septic Cardiomyopathy via Bioinformatics Analysis

**DOI:** 10.3389/fgene.2022.827559

**Published:** 2022-04-13

**Authors:** Cheng-Wu Gong, Ming-Ming Yuan, Bai-Quan Qiu, Li-Jun Wang, Hua-Xi Zou, Tie Hu, Song-Qing Lai, Ji-Chun Liu

**Affiliations:** ^1^ Department of Cardiothoracic Surgery, The Second Affiliated Hospital of Nanchang University, Nanchang, China; ^2^ Department of Cardiothoracic Surgery, The First Affiliated Hospital of Nanchang University, Nanchang, China; ^3^ Institute of Cardiovascular Diseases, Jiangxi Academy of Clinical Medical Sciences, The First Affiliated Hospital of Nanchang University, Nanchang, China

**Keywords:** septic cardiomyopathy, ferroptosis, miRNA, mRNA, public database

## Abstract

Septic cardiomyopathy (SCM) is a cardiac dysfunction caused by severe sepsis and septic shock that increases the risk of heart failure and death and its molecular mechanism remains unclear. Ferroptosis, a novel form of programmed cell death, has been reported to be present in the heart tissue of patients with sepsis, which demonstrated that ferroptosis may be a potential mechanism of myocardial injury in SCM. Therefore, we explored the role of ferroptosis-related genes (FRGs) in SCM and aimed to identify pivotal ferroptosis-related targets in SCM and potential therapeutic targets involved in the pathological process of SCM. To explore the regulatory mechanisms of ferroptosis in SCM, we identified differentially expressed genes (DEGs) in SCM and FRGs by bioinformatics analysis, and further identified hub genes. And the crucial microRNAs (miRNAs)-FRGs regulatory network was subsequently constructed. Finally, several candidate drugs associated with the hub genes were predicted, and Real-time quantitative reverse Transcription PCR (qRT-PCR) and western blotting analysis were performed to confirm the abnormal expression of hub genes. In this study, we identified several FRGs that may be involved in the pathogenesis of SCM, which helps us further clarify the role of ferroptosis in SCM and deeply understand the molecular mechanisms and potential therapeutic targets of SCM.

## Introduction

Sepsis, a syndrome of physiologic, pathologic, and biochemical abnormalities induced by infection (Cecconi et al., 2018), has been identified by the World Health Organization as a global healthy priority (Reinhart et al., 2017). Septic cardiomyopathy (SCM) is a significant morbid component of severe sepsis and septic shock (Balija and Lowry 2011). Myocardial cell injury and cardiomyocyte contractile dysfunction are the main pathophysiological processes in SCM, and clinical diagnosis can only rely on myocardial damage factors and echocardiography, which limits early intervention in patients with SCM (Crouser 2004; Rudiger and Singer 2007; Hassoun et al., 2008; Werdan et al., 2009; Celes et al., 2010). Therefore, it is crucial to select appropriate predictive biomarkers for early intervention in SCM.

MiRNAs, small, single-stranded, non-coding RNAs (18–24 nucleotides), well conserved in eukaryotic organisms, play biological roles in inflammation, metabolism, and development (Mendell and Olson 2012; Manetti et al., 2021). What’s more, in inflammatory heart diseases and sepsis-induced cardiac dysfunction, circulating miRNAs were proposed as biomarkers for diagnosis and disease monitoring (Mirna et al., 2019; Manetti et al., 2020; Chiti et al., 2021). With the development of high-throughput gene expression profiling technology, Microarray analysis has been developed as detection technology to simultaneously monitor the differential expression of numerous genes or miRNAs in various studies, including in the field of SCM (Vogelstein et al., 2013; Zhang et al., 2019a; Reyes et al., 2020).

**FIGURE 1:**
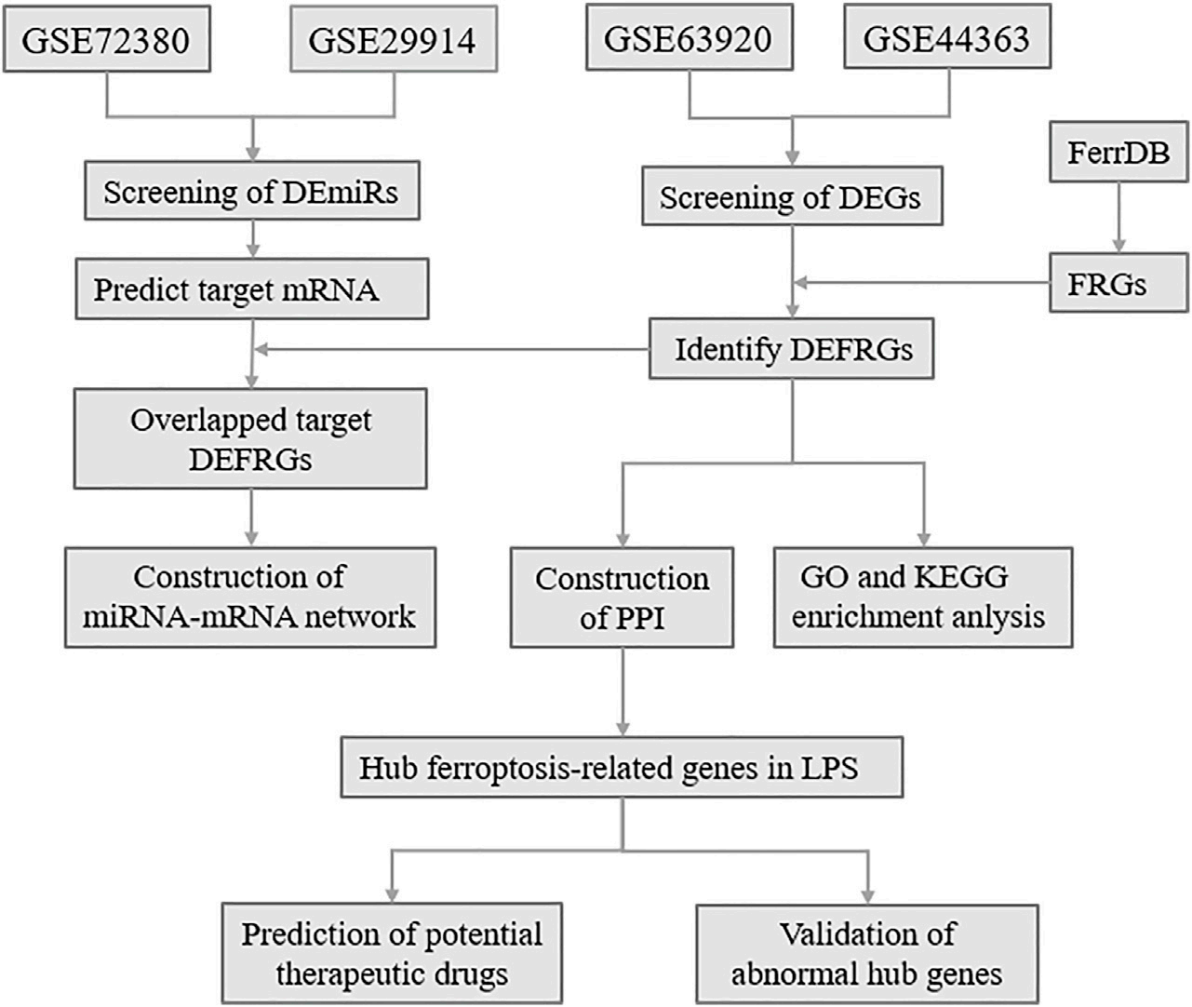
The overall protocol of this study. DEmiRs, differentially expressed microRNA; DEmRs, differentially expressed mRNA; FRGs, ferroptosis-related genes; DEFRGs, differentially expressed FRGs; PPI, protein-protein interaction.

Ferroptosis is an iron-dependent, novel form of programmed cell death that differs from apoptosis, cell necrosis, and autophagy (Tang et al., 2021). Ferroptosis was proposed in 2012 by Dixon, S.J. et al. (Dixon et al., 2012), and is characterized essentially by iron-dependent lipid peroxidation. Ferroptosis is mainly related to pathological cell death associated with mammalian degenerative diseases, carcinogenic effects, cerebral hemorrhage, local ischemia-reperfusion injury, and kidney degeneration (Stockwell et al., 2017; Fang et al., 2020). Wang et al. (Wang et al., 2020) reported that the reduction of ferroptosis via enhancing GPX4, may be the major mechanisms via which Dexmedetomidine alleviates sepsis-induced myocardial cellular injury. It is suggested that ferroptosis may be an important part of myocardial injury in SCM. However, the exact mechanism of ferroptosis in LPS-induced myocardial injury remains unclear. Here, to determine the role of ferroptosis in SCM, we identified FRGs based on relevant databases and analyzed the differential expression of FRGs among SCM and normal samples, and further identified differentially expressed FRGs (DEFRGs) by bioinformatic analysis. GO and KEGG Pathway enrichment analysis of DEFRGs were performed subsequently. After screening out the differentially expressed miRNAs (DEmiRs), we obtained the target mRNAs of DEmiRs from the relevant databases. The crucial miRNAs-FRGs regulatory network was subsequently constructed. Based on the STRING online database, we established a protein-protein interaction (PPI) network, and Hub genes were screened out from PPI network, and further identified potential therapeutic drugs of SCM by DSigDB database. Furthermore, qRT-PCR and Western Blotting Analysis were performed to confirm the abnormal expression of hub genes. The flow chart of the whole study was shown in Figure 1.

## Materials and Methods

### Data Collection

The microarray data were obtained from GEO database (http://www.ncbi.nlm.nih.gov/geo/). Two miRNA expression profiles (GSE72380 and GSE29914) and two mRNA expression profiles (GSE63920 and GSE44363) between LPS and Saline were downloaded and investigated. The FRGs were identified from FerrDb (https://www.zhounan.org/ferrdb) and GeneCards (https://www.genecards.org). After deduplication of genes, the merged FRGs set contains 442 FRGs, listed in Supplementary Table S1. The GSE72380 dataset was comprised of 6 mice hearts tissue treated with LPS and 6 mice hearts tissue treated with saline (Wang et al., 2016); GSE29914 contained 4 LPS treated mice hearts tissue and 4 saline treated mice hearts tissue sample miRNA expression profiles (Drosatos et al., 2011); GSE63920 included 4 LPS treated mice hearts tissue and 4 saline treated mice hearts tissue sample mRNA expression profiles (Drosatos et al., 2016); and GSE44363 contained 24 mice hearts tissue samples, including 4 LPS samples and 4 saline samples. All datasets were downloaded in a processed and normalized format.

### Data Processing

The R-based GEO online tool GEO2R (http://www.ncbi.nlm.nih.gov/geo/geo2r/) was used to identify the differentially expressed miRNAs (DEmiRs) and mRNAs (DEmRs) between LPS and saline treated mice hearts tissue expression profiles, with the thresholds of |log2 (fold-change)| > 1.0 and adjusted *p*-value < 0.05.

### Functional and Pathway Analysis

Database for Annotation, Visualization, and Integrated Discovery (DAVID) (https://david.ncifcrf.gov/) was used to perform Gene Ontology (GO) and Kyoto Encyclopedia of Genes and Genomes (KEGG) analysis with DEFRGs. *p* < 0.05 was applied as the cut-off criterion.

### Construction of Protein-Protein Interaction Network and Identification of Hub Genes

The STRING Database (Szklarczyk et al., 2015) (https://string-db.org/) is a flexible and user-friendly website for generating hypotheses about gene function, analyzing gene lists, and prioritizing genes for functional assays. In this study, the network of DEFRGs was constructed using the STRING Database. Minimum required interaction score was selected as medium confidence (0.4). In addition, cytoHubba app of Cytoscape was used to determine hub genes. The plot displayed the ranking of the 10 molecules by the shade of each color: The darkest red marked the first, the lightest yellow marked the last.

### Prediction of the Target Genes of Differentially Expressed miRs and Construction miRNA-mRNA Networks

The target mRNAs of DEmiRs were obtained from TargetScan (www.targetscan.org/) (Fehlmann et al., 2019) and miRDB (http://www.mirdb.org/) (Wong and Wang 2015). And the Overlapped target DEFRGs between predicted mRNAs and DEFRGs were identified. And the miRNA-mRNA pairs were identified based on the connection between DEmiRs and DEFRGs. Then, the miRNA-mRNA pairs were imported into Cytoscape software to construct the initial miRNA-mRNA network. Finally, the miRNA-mRNA interaction network was depicted and visualized using cytoscape 3.8.2 software.

### Drug Prediction of Differentially Expressed mRs

The DSigDB database (http://dsigdb.tanlab.org/) contains 22 527 gene sets, consists of 17 389 unique compounds covering 19 531 genes The DSigDB database aids the identification of novel drug targets and the comparison of drug structures with potential mechanisms of action (Yoo et al., 2015). The hub genes were input into DSigDB database to examine their association with potential targeted drugs.

### Construction of the Cardiomyocyte Injury Model

To verify our results, we constructed an LPS-induced myocardial injury model of SCM. Lipopolysaccharide (LPS) was purchased from Sigma (United States); ferrostatin-1 (Fer-1) (≥99.72% purity) was obtained from Solarbio (Shanghai, China). HL-1 cells (a cardiac muscle cell line that contracts and retains phenotypic characteristics of the adult cardiomyocyte) were provided by the Cell Bank of the Chinese Academy of Sciences. Cells were cultured in MEM Alpha Modification containing 10% fetal bovine serum (FBS). LPS (5 μg/ml) was added into the cell culture medium to induce an *in vitro* cardiomyocyte injury model. In addition, to further increase the confidence of the results, we used Cdkn1a small interfering RNA (Si-Cdkn1a) to validate our predicted results. HL-1 cells were transfected with Si-Cdkn1a by riboFECT™ CP Reagent (RIBOBIO, China) according to protocol for 24 or 36 h. HL-1 cells were pretreated with Fer-1 (1 μmol/L) for 1 h before LPS stimulation.

### Real-Time Quantitative Reverse Transcription PCR

Total RNA was isolated from HL-1 cells with Trizol reagent (Invitrogen, CA, United States), and was reverse transcribed into cDNA using Revert Aid First Strand cDNA Synthesis Kit (Thermo Fisher Scientific, Shanghai, China). Real-time PCR assays were performed using SYBR rapid quantitative PCR Kit (Thermo Fisher Scientific, Shanghai, China). The mRNA and miRNA primers were designed and synthesized by The Beijing Genomics Institute (BGI). The primer sequences were supplemented in Table 1. The target genes were quantified relative to internal control using 2^-△CT^ algorithm (GAPDH/U6), and the differences between the different groups were confirmed.

**TABLE 1:** The primer sequences of hub genes.

Primer	Sequence
GAPDH-F	GGT​CGG​TGT​GAA​CGG​ATT​T
GAPDH-R	TGA​ACT​TGC​CGT​GGG​TAG​A
Cdkn1a-F	GCA​AAG​TGT​GCC​GTT​GTC​TC
Cdkn1a-R	CGT​CTC​CGT​GAC​GAA​GTC​AA
Vim-F	TTC​TCT​GGC​ACG​TCT​TGA​CC
Vim-R	CTT​TCA​TAC​TGC​TGG​CGC​AC
Rela-F	CAA​GAC​ACA​CCC​CAC​CAT​CA
Rela-R	CTC​TAT​AGG​AAC​TAT​GGA​TAC​TGC​G
Arntl-F	GAT​CGC​GGA​GGA​AAT​CAT​GG
Arntl-R	TGA​GCC​TGC​CCT​GGT​AAT​AG
Nfe2l2-F	AGC​CAG​CTG​ACC​TCC​TTA​GA
Nfe2l2-R	AGT​GAC​TGA​CTG​ATG​GCA​GC
Socs1-F	CAA​CGG​AAC​TGC​TTC​TTC​GC
Socs1-R	AGC​TCG​AAA​AGG​CAG​TCG​AA
Slc39a14-F	GAT​ATC​GGG​ACC​TTG​GCC​TG
Slc39a14-R	AGA​GAA​TGG​CCA​CTG​ACG​TG
Ptgs2-F	GGT​GCC​TGG​TCT​GAT​GAT​GTA​TGC
Ptgs2-R	GGA​TGC​TCC​TGC​TTG​AGT​ATG​TCG

### Western Blotting Analysis

Total protein from HL-1 cells was extracted using RIPA lysis buffer. Proteins in the samples (20 μg) were separated by 12% SDS-PAGE and subsequently transferred to PVDF membranes. The membranes were then incubated with 5% non-fat milk for 1 h at room temperature to block nonspecific binding and incubated with primary antibodies against GPX4 (1:1,000, ZENBIO, chain), FTH1 (1:1,000, ZENBIO, chain), Caspase-3 (1:1,000, Abcam), Bcl-2 (1:5,000, Proteintech), Cdkn1a (Proteintech), Bax (1:2,000, cell Signaling Technology),, and *β*-actin (1:1,000, Abcam) at 4 °C overnight, followed by incubation with goat anti-rabbit HRP-conjugated secondary antibody (1:5,000, Abcam) at room temperature for 1 h. Protein bands were visualized using an enhanced chemiluminescence kit (Beyotime, China), and *β*-actin was used as an internal control for the relative protein expression. Protein bands were quantified using the ImageJ software.

### Statistical Analysis

Data are presented as the mean ± SEMs or SDs. Statistical analyses were performed in GraphPad Prism 7 (GraphPad Software, Inc.) using unpaired two-tailed Student’s t-test to compare differences between two groups with significance of *p* < 0.05. One-way analysis of variance with multiple comparisons tests was used to compare three or more groups with significance of *p* < 0.05.

## Results

### Identification of Differentially Expressed miRs and Differentially Expressed mRs

**FIGURE 2:**
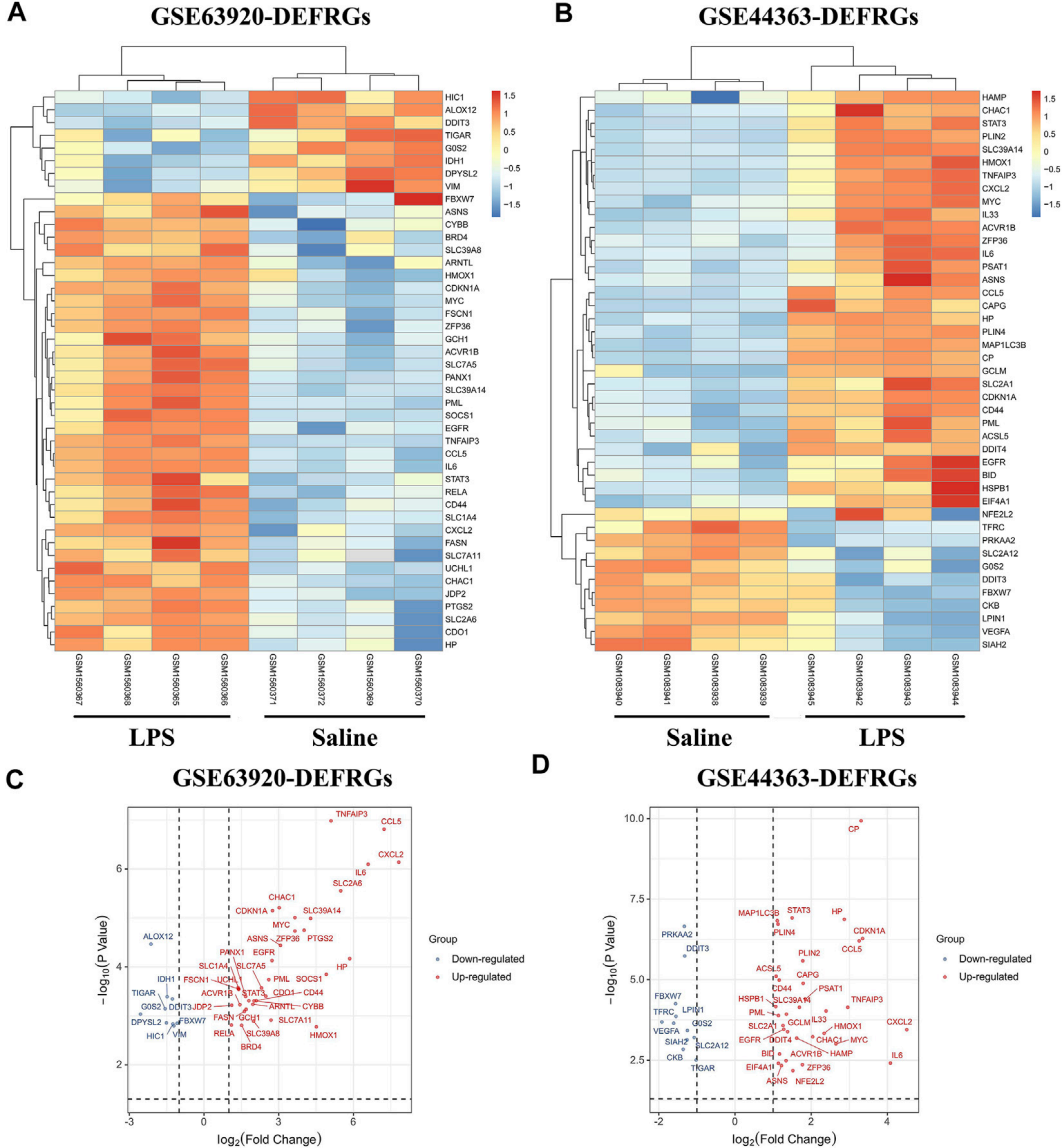
The heatmaps and volcano plots of DEFRGs. **(A)** The heatmap of DEFRGs in GSE63920; **(B)** The heatmap of DEFRGs in GSE44363; **(C)** The volcano plot of DEFRGs in GSE63920; **(D)** The volcano plot of DEFRGs in GSE44363. FRGs, ferroptosis-related genes; DEFRGs, differentially expressed FRGs.

**FIGURE 3:**
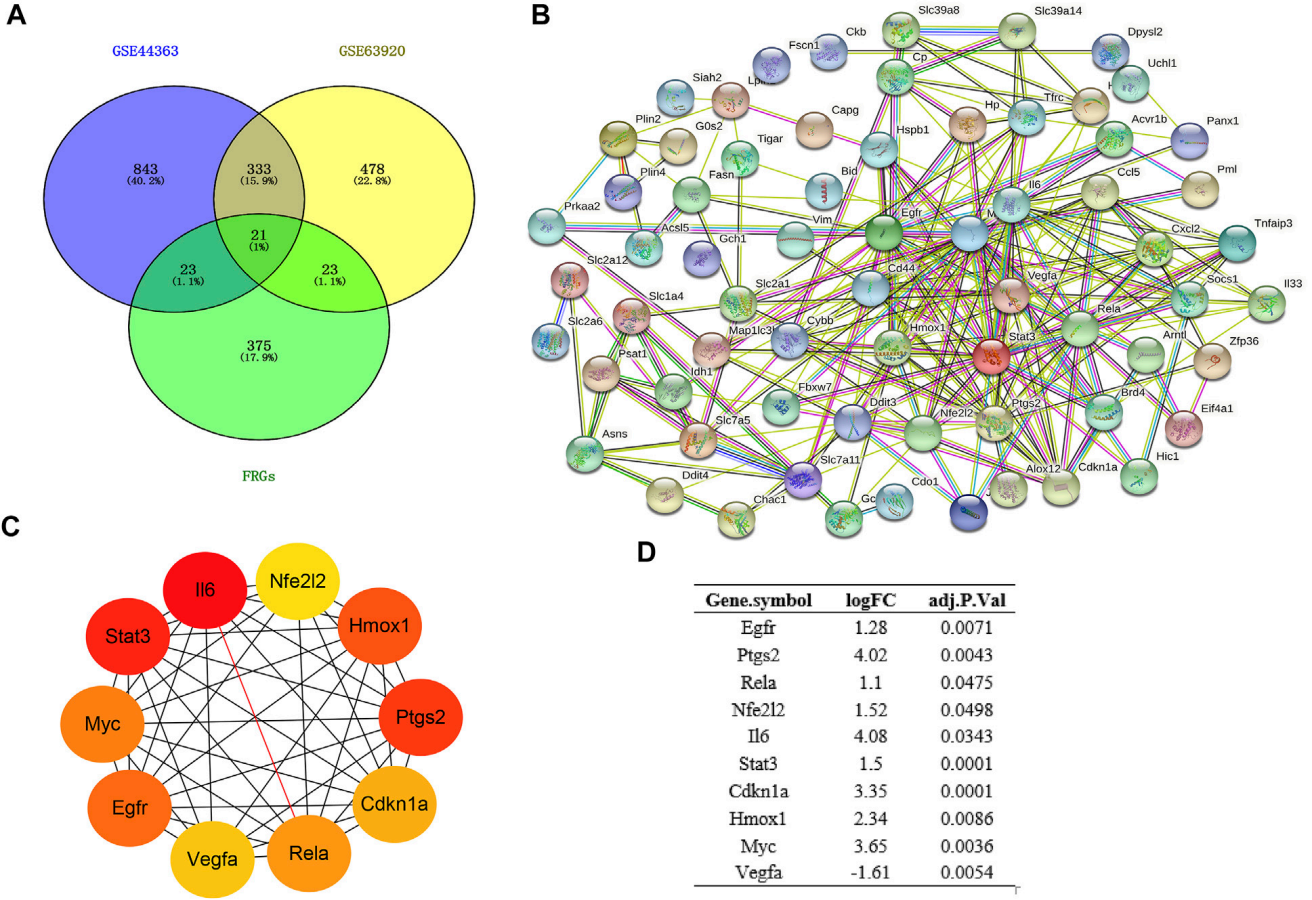
Construction of PPI network and Identification of hub genes. **(A)** Venn diagram of DEGs between DEmRs and FRGs; **(B)** PPI network of DEFRGs constructed by STRING database; **(C)** the top 10 hub genes were identified by MCC; **(D)** the logFC and adjusted *p*-value of the hub genes. DEGs, differentially expressed genes; FRGs, ferroptosis-related genes; DEFRGs, differentially expressed FRGs.

The total number of miRNAs were 1,200 and 318 from GSE72380 and GSE29914, while the total number of mRNAs were 34945 and 45101 from GSE63920 and GSE44363, respectively (Table 2). According to the predetermined threshold (|log2FC| > 1.0 and adjusted *p* < 0.05), we identified 2 DEmiRs (2 upregulated) from GSE72380 dataset and 6 DEmiRs (3 upregulated and 3 downregulated) from GSE29914 dataset (Table 3, Supplementary Tables S2–S3). Also, 1,206 DEmRs (878 upregulated and 328 downregulated) in GSE63920 and 1,690 DEmRs (901 upregulated and 789 downregulated) in GSE44363 were identified **(** Supplementary Tables S4–S5 **)**. Then, a heatmap of the top 25 DEmiRs and DEmRs were constructed (Supplementary Figure S1 **)**, while the volcano plots of DEmiRs and DEmRs are shown in Supplementary Figure S2. Finally, 67 overlapping DEFRGs were found between DEGs and FRGs (Figure 3A). Subsequently, the heatmaps and volcano plots of DEFRGs in dataset GSE44363 and GSE63920 were constructed, respectively (Figure 2).

**TABLE 2:** Characteristics for GEO microarray in LPS hearts tissues.

Data Sourse	Type	Sourse	LPS/Control	Platform	Annotation of Platform
GSE72380	miRNA	heart samples	6/6	GPL19324	Agilent-038112 mouse miRNA microarray, Release 18.0 (miRNA ID version)
GSE29914	miRNA	hearts samples	4/4	GPL13715	ORB Sanger15 Multispecies MIcroRNA Microarray
GSE63920	mRNA	hearts samples	4/4	GPL19507	MI mouse exonic evidence-based oligonucleotide (MEEBO) microarray
GSE44363	mRNA	hearts samples	4/4	GPL1261	[Mouse430_2] Affymetrix Mouse Genome 430 2.0 Array

GEO, Gene expression omnibus; LPS, Lipopolysaccharide; miRNA, microrna; Mrna, messenger RNA.

**TABLE 3:** Key DE miRs accessed from GSE72380 and GSE29914.

miRNA_ID	Log FC	*p* Value
mmu-miR-21-3p	5.018872	3.19E-07
mmu-miR-155-5p	1.056015	4.70E-05
mmu-miR-2132	1.18572	0.00656
mmu-miR-155	1.264	0.00757
mmu-miR-125b-3p	−1.89198	0.02283
mmu-miR-1892	−1.02102	0.03988
mmu-miR-2137	1.23337	0.03988
mmu-miR-341	−1.69248	0.04126

### Establishment of Protein-Protein Interaction Network and Identification of Hub Genes

Based on the STRING database, the visual PPI network of DEFRGs was constructed (Figure 3B). Furthermore, the STRING database analysis showed that these genes were significantly associated with oxidoreductase activity. In the PPI network, some key nodes are closely linked and the Cytohubba plugin of Cytoscape software was used to screen these hub genes. Then, ten hub genes were screened out with MCC algorithms, including Egfr, Ptgs2, Cdkn1a, Nfe2l2, Rela, Il6, Hmox1, Stat3, Myc and Vegfa (Figure 3C). And Figure 3D shows the logFC and adjusted *p*-value of the hub genes.

### Gene Ontology and Kyoto Encyclopedia of Genes and Genomes Pathway Enrichment Analysis of Differentially Expressed FRGs

**FIGURE 4:**
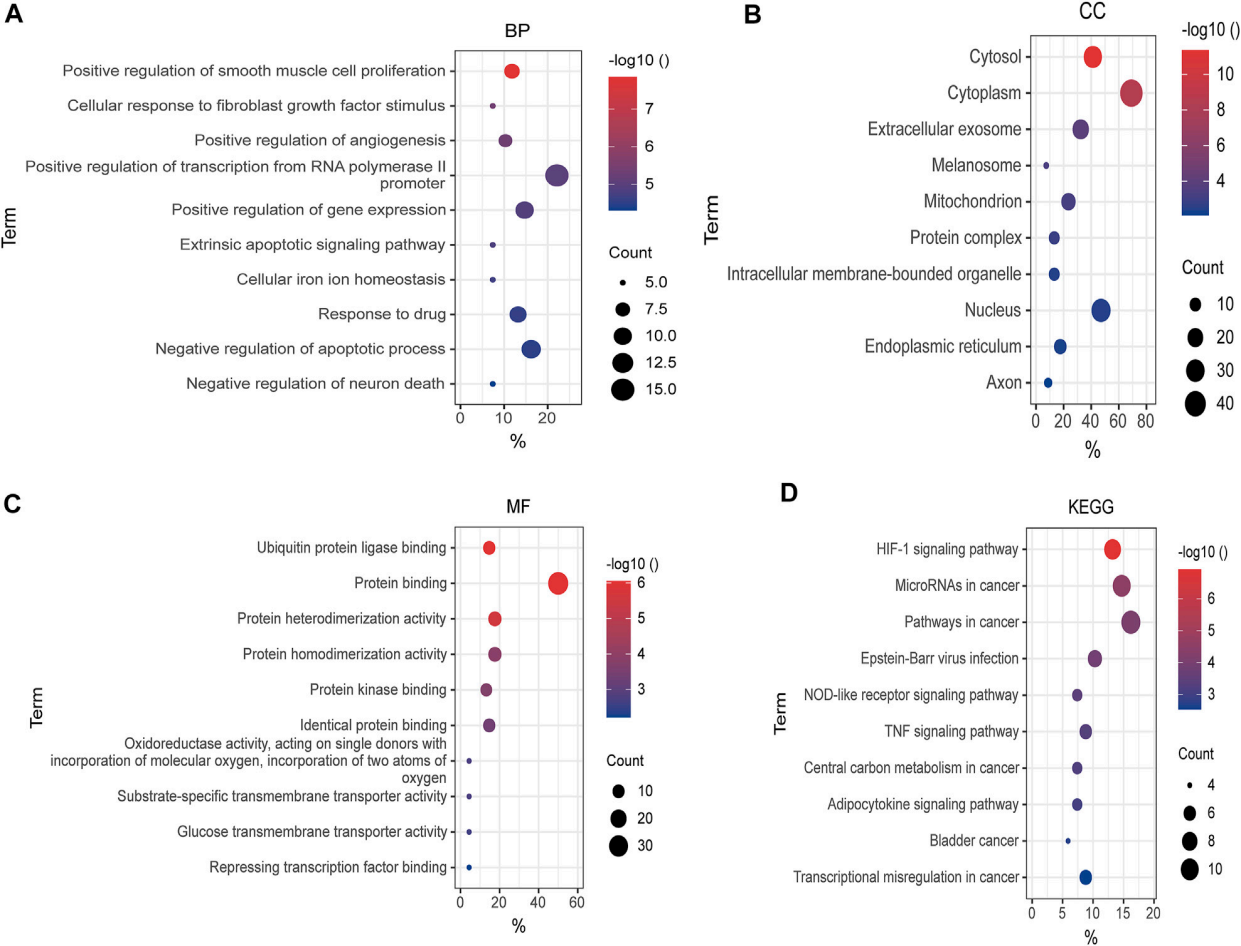
GO and KEGG enrichment analysis of DEFRGs. **(A)** GO enrichment analysis of DEFRGs in the biological process category (BP); **(B)** the cellular component category (CC); **(C)** the molecular function category (MF); **(D)** KEGG enrichment analysis of DEFRGs. DEFRGs, differentially expressed FRGs.

To make a thorough understanding of the function of DEFRGs, GO and KEGG Pathway enrichment analysis of DEFRGs were performed. These duplicated functions or pathways present in both results were shown below. Biological processes (BP) included “positive regulation of smooth muscle cell proliferation”, “cellular response to fibroblast growth factor stimulus”, “positive regulation of angiogenesis”, “positive regulation of transcription from RNA polymerase II promoter”, and “positive regulation of gene expression” (Figure 4A). Besides, “cytosol”, “cytoplasm”, “extracellular exosome”, “melanosome”, “mitochondrion” and “protein complex” were contained in Cellular Components (Figure 4B), while Molecular Functions (MF) included “ubiquitin protein ligase binding”, “protein binding”, “protein heterodimerization activity”, “protein homodimerization activity”, “protein kinase binding” and “identical protein binding” (Figure 4C). In addition, these genes were both significantly associated with 6 KEGG pathways, including “HIF-1 signaling pathway”, “MicroRNAs in cancer”, “Pathways in cancer”, “Epstein-Barr virus infection”, “NOD-like receptor signaling pathway” and “TNF signaling pathway”. (Figure 4D).

### Prediction of the Target Genes of Differentially Expressed miRs and Construction miRNA-mRNA Networks

**FIGURE 5:**
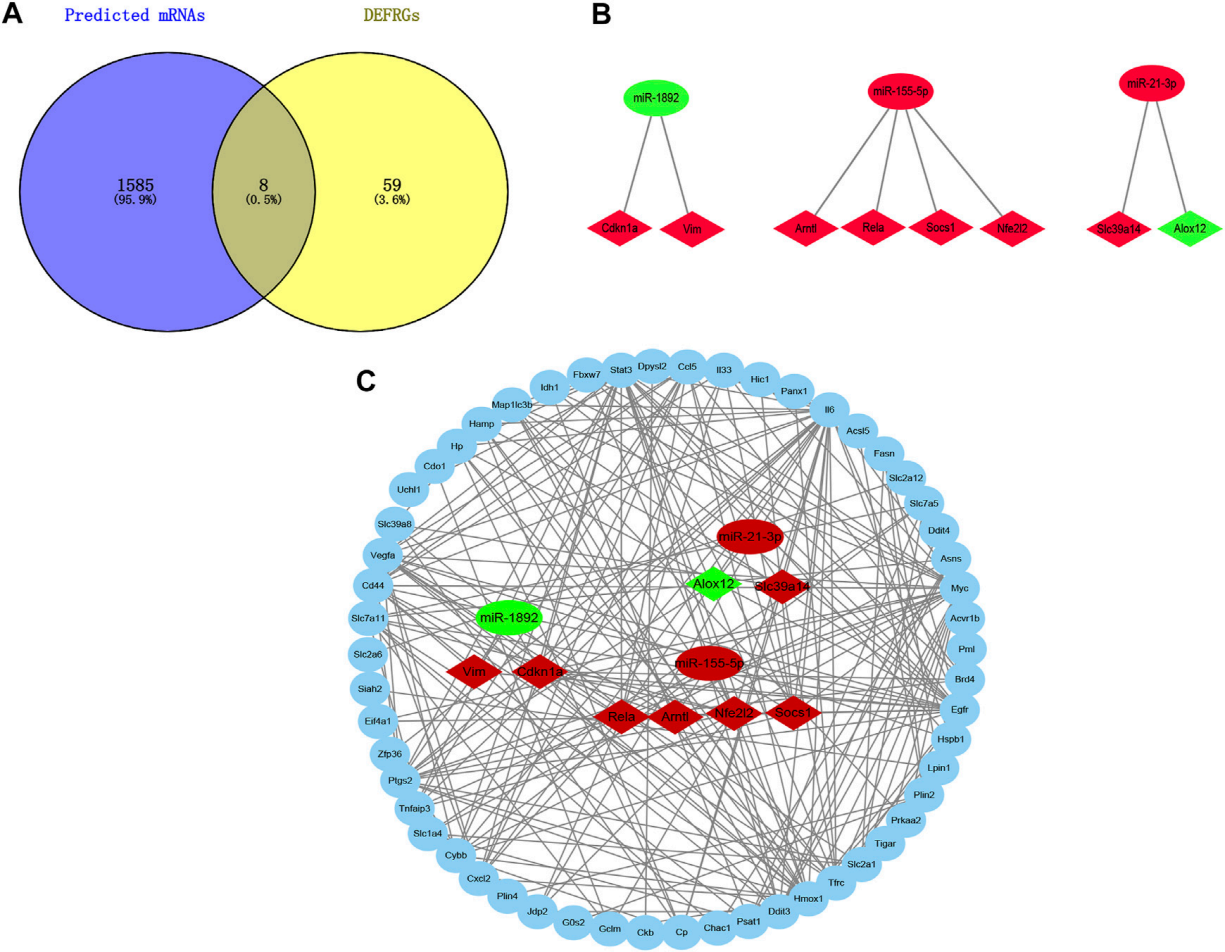
Construction of DEmiRs-DEFRGs regulatory network. **(A)** Venn diagram showing the overlap of genes between predicted mRNAs and DEFRGs. **(B)** The miRNA- DEFRGs regulatory pairs. **(C)** The miRNA- DEFRGs regulatory network. DEFRGs, differentially expressed ferroptosis-related genes.

The target mRNAs of those 8 DEmiRs were gained from two miRNA target prediction websites (TargetScan and miRDB). A total of 1,593 predicted mRNAs were subsequently identified. And 8 Overlapped target DEFRGs between predicted mRNAs and DEFRGs were found, including Cdkn1a, Nfe2l2, Rela, Vim, Arntl, Socs1, Slc39a14 and Alox12. (Figure 5A). Next, based on the association between DEmiRs and the DEFRGs, the miRNA- DEFRGs pairs were identified (Figure 5B). Subsequently, we merged the miRNA- DEFRGs pairs and PPI network into the final miRNA-mRNA network by using Cytoscape software (Figure 5C).

### Drug Prediction of Differentially Expressed mRs

**FIGURE 6:**
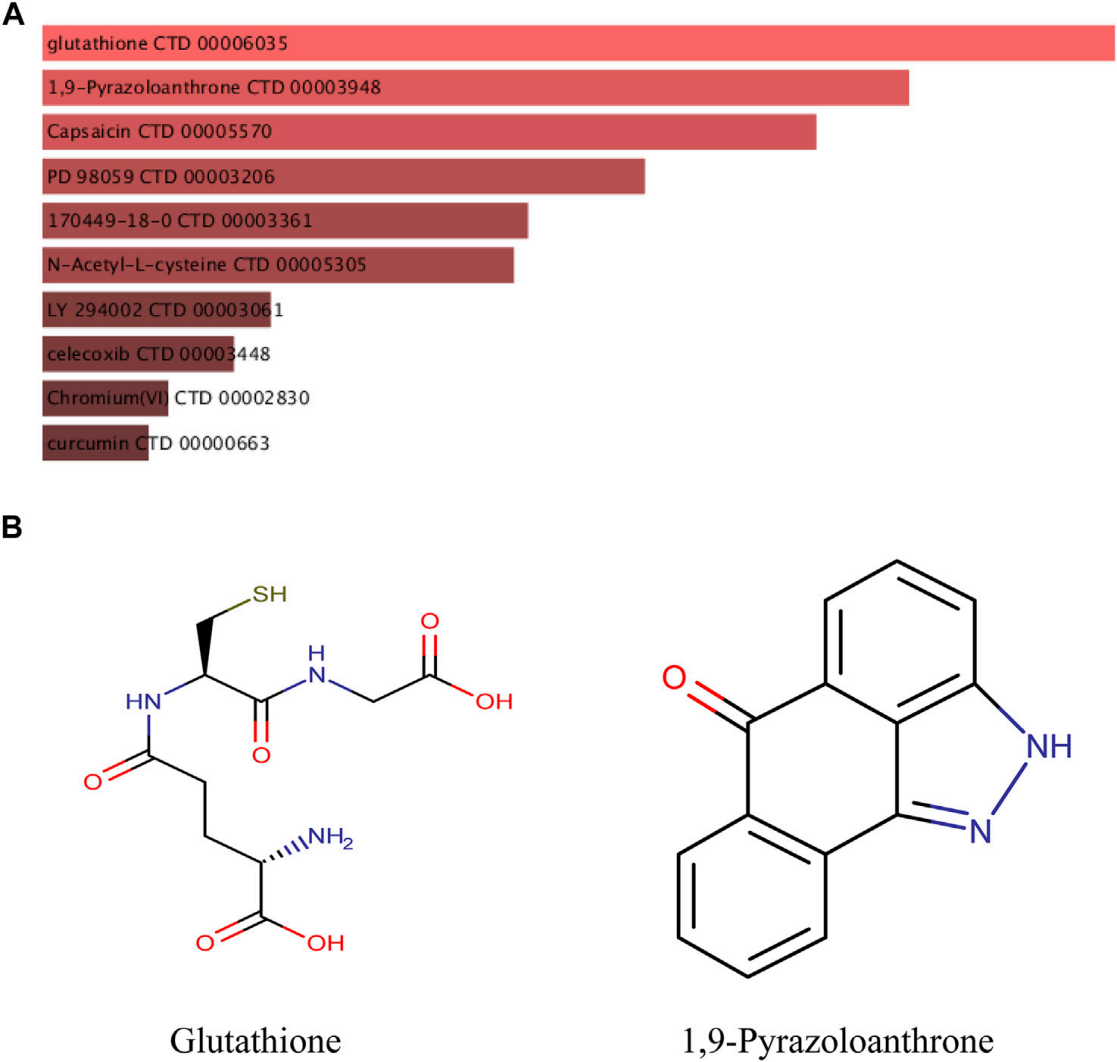
Targeted drugs prediction. **(A)** The top 10 predicted target drugs ranked according to *p* values in the DSigDB database. **(B)** The chemical structures of glutathione and 1,9-Pyrazoloanthrone.

The DSigDB database can combine detailed drug data with comprehensive drug targets and drug action information. Its main functions included promotion of silicon-chip drug target discovery, drug design, drug docking or screening, drug metabolism prediction, drug interaction prediction, and general pharmacy education. The 10 hub genes were inserted into DSigDB database, and the targeted drugs of these genes were predicted. The top 10 candidate drugs related to the DEmRs were chosen according to the *p*-value and adjusted *p*-value and all these drugs have been approved by Food and Drug Administration or have been experimental stage (Table 4 **)**. The 10 drugs were shown in Figure 6A and the structure of the top two drugs were shown in Figure 6B. The effects of these drugs were consistent with the international guidelines for management of sepsis (29).

**TABLE 4:** The predicted drugs of DEmRs.

Term	Groups	*p*-Value	Adj *p*-value	Genes
glutathione	Approved	1.51E-22	3.63E-19	IL6; CDKN1A; MYC; STAT3; HMOX1; PTGS2; EGFR; RELA; NFE2L2; VEGFA
1,9-Pyrazoloanthrone	Experimental	2.86E-21	3.45E-18	IL6; CDKN1A; MYC; STAT3; HMOX1; PTGS2; EGFR; RELA; NFE2L2; VEGFA
Capsaicin	Approved	1.08E-20	8.66E-18	IL6; CDKN1A; MYC; STAT3; HMOX1; PTGS2; EGFR; RELA; NFE2L2; VEGFA
PD 98059	Approved	1.25E-19	7.55E-17	IL6; CDKN1A; MYC; STAT3; HMOX1; PTGS2; EGFR; RELA; NFE2L2; VEGFA
170,449–18-0	Experimental	6.66E-19	3.21E-16	IL6; CDKN1A; STAT3; HMOX1; PTGS2; EGFR; NFE2L2; VEGFA
N-Acetyl-L-cysteine	Approved, Investigational	8.14E-19	3.27E-16	IL6; CDKN1A; MYC; STAT3; HMOX1; PTGS2; EGFR; RELA; NFE2L2; VEGFA
			
LY 294002	Experimental	2.64E-17	9.09E-15	IL6; CDKN1A; STAT3; HMOX1; PTGS2; EGFR; RELA; NFE2L2; VEGFA
Celecoxib	Approved	4.46E-17	1.34E-14	IL6; CDKN1A; MYC; STAT3; PTGS2; RELA; NFE2L2; VEGFA
	Investigational			
Chromium	Approved	1.14E-16	3.05E-14	IL6; MYC; STAT3; HMOX1; PTGS2; EGFR; RELA; VEGFA
curcumin	Approved	1.51E-16	3.51E-14	IL6; CDKN1A; MYC; STAT3; HMOX1; PTGS2; EGFR; RELA; NFE2L2; VEGFA

### Validation of Abnormal Hub Genes

**FIGURE 7:**
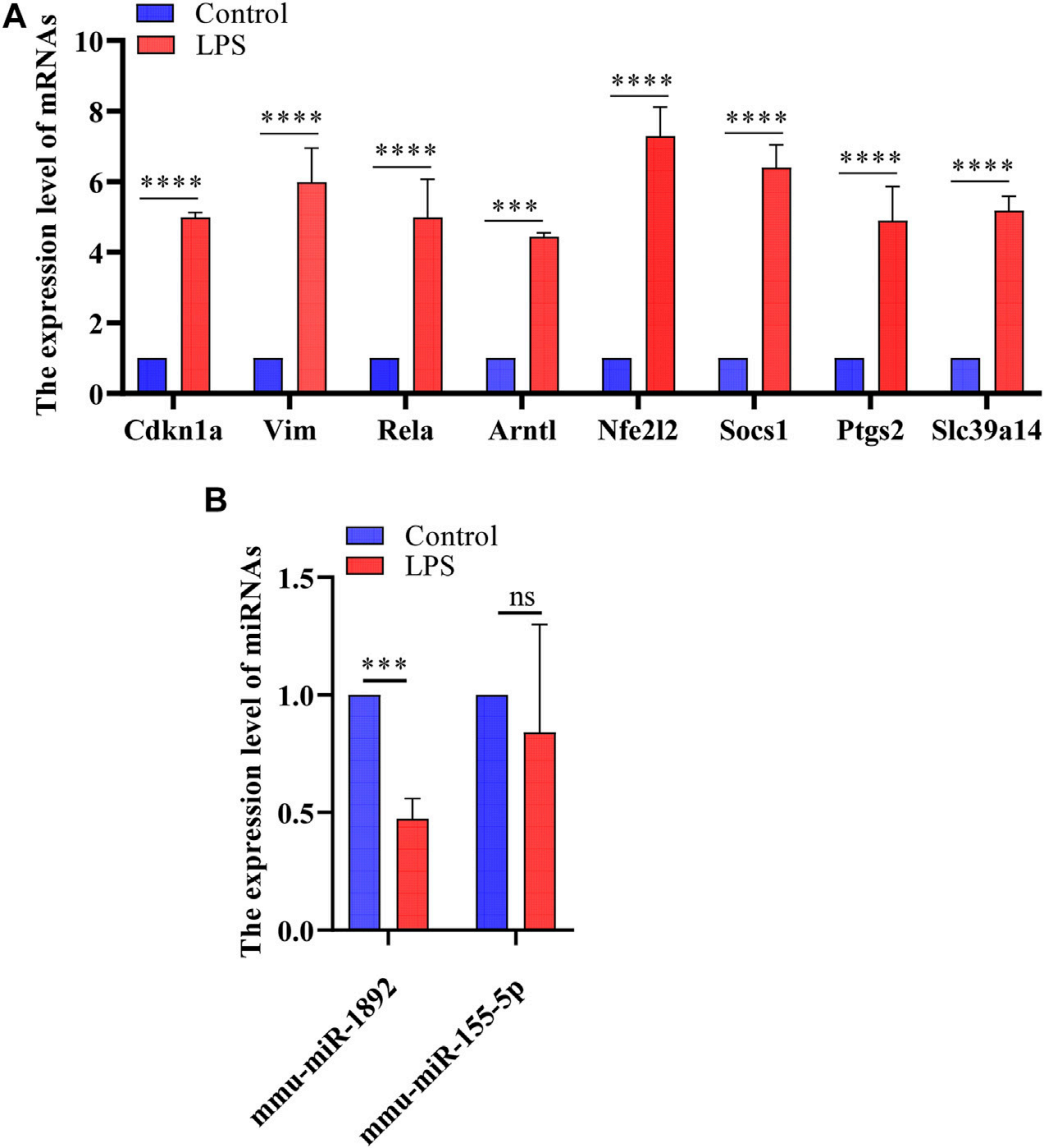
The results of real-time PCR. **(A)** Detection of FRGs expression in control and LPS treat HL-1 cells using RT-qPCR (n = 3), compared with the control group, the expression of FRGs was upregulated in HL-1 cells treated with LPS. **(B)** Detection of miRNAs expression using RT-qPCR (*n* = 3), the expression of mmu-miR-1892 was significantly down-regulated in LPS-treated HL-1 cells. ***, *p* < 0.001; ****, *p* < 0.0001; ns, no significant. Error bars represent the mean ± SD of triplicate experiments.

**FIGURE 8:**
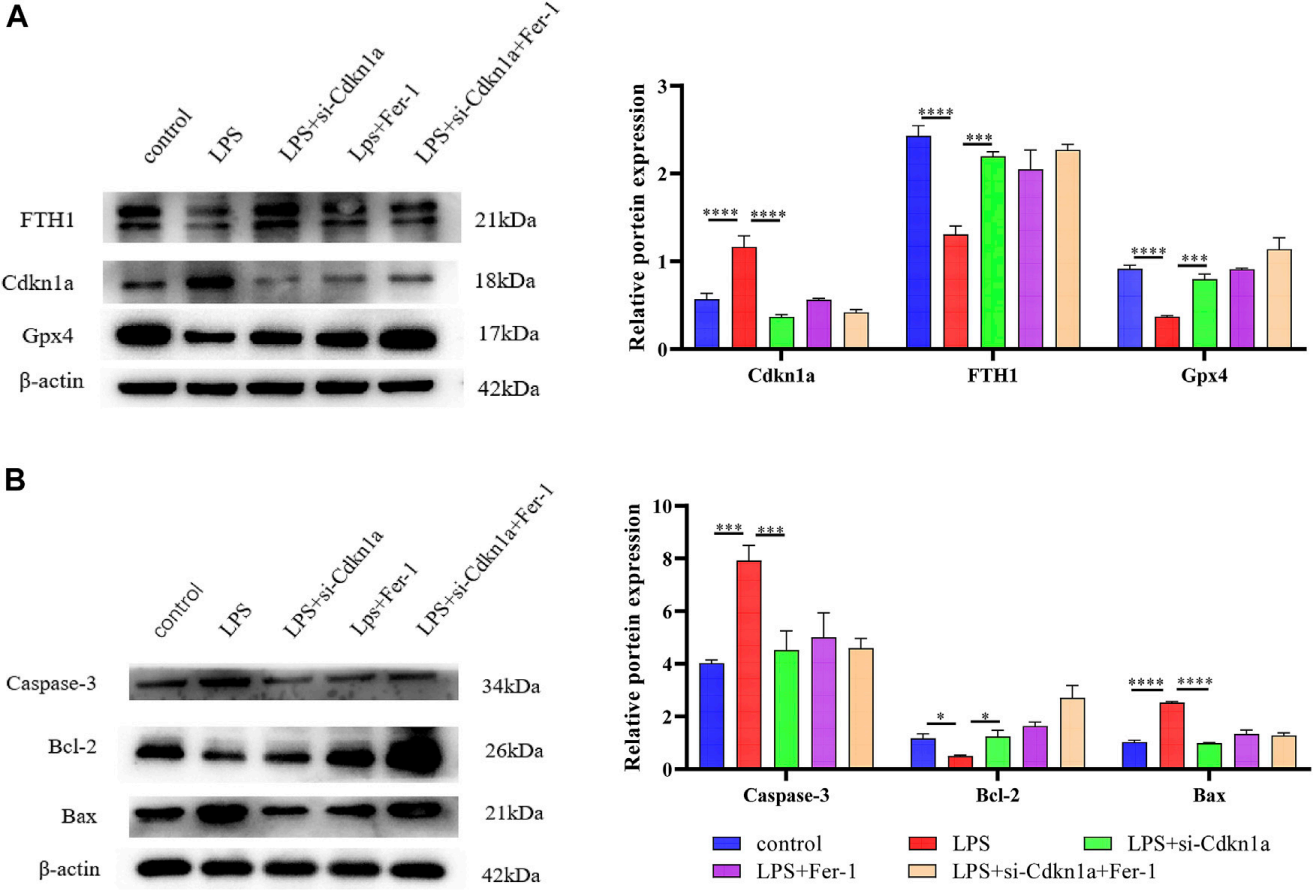
The results of Western Blotting Analysis. This part of the study was based on the untreated HL-1 cells and LPS-treated HL-1 cells that were treated with Si- Cdkn1a or Fer-1 (*n* = 3). **(A)** Western blotting was used for detecting GPX4, FTH1 and Cdkn1a expression in different HL-1 cells; **(B)** Western blotting was used for detecting Caspase-3, Bcl-2 and Bax expression in different HL-1 cells. *, *p* < 0.05; ***, *p* < 0.001; ****, *p* < 0.0001, Error bars represent the mean ± SD of triplicate experiments.

Based on the miRNA–mRNA network, we validated several hub genes to increase the confidence of our results. We constructed an LPS-induced myocardial injury model of SCM and determined the expression of differential mRNAs using real-time PCR. The results are shown in Figure 7. Compared with the control group, the expression of FRGs Cdkn1a, Vim, Rela, Arntl, Nfe2l2, Socs1, Ptgs2 and Slc39a14 were upregulated in HL-1 cells treated with LPS (Figure 7A). In addition, the expression of mmu-miR-1892 was significantly down-regulated in LPS-treated HL-1 cells (Figure 7B). These findings were consistent with our predicted results. Based on the above results, we selected mmu-miR-1892 -Cdkn1a, a regulatory pair, for further protein-level validation. To verify the protein levels of Cdkn1a and to further explore the effect of Cdkn1a on ferroptosis in SCM, we performed western blotting analysis. As shown in Figure 8A, the expression of Cdkn1a was up-regulated in HL-1 cells treated with LPS, but the expression of Gpx4 and FTH1, ferroptosis-related proteins, were down-regulated in LPS-treated HL-1 cells, however, Si- Cdkn1a treatment reversed this discrepancy, suggesting that Cdkn1a may be a potential regulatory target of LPS-induced ferroptosis in cardiomyocytes. Furthermore, to further explore the effect of Cdkn1a on LPS-induced myocardial injury, we simultaneously examined the levels of apoptosis-related proteins. As shown in Figure 8B, the expression of Caspase-3 and Bax were significantly up-regulated, but the expression of Bcl-2 was down-regulated in LPS-treated HL-1 cells. Interestingly, Si- Cdkn1a inhibited the expression of apoptosis-associated proteins and increased the expression of Bcl-2, indicating that Cdkn1a may be inhibiting LPS-induced cardiomyocyte injury through diverse pathways, and the specific mechanism of action needs to be further studied.

## Discussion

Sepsis is a manifestation of the body’s dysfunctional response to infection, which may eventually lead to multi-organ failure, disability and even death (Deutschman and Tracey 2014). SCM is a serious complication of sepsis and is directly associated with high sepsis mortality (Liu et al., 2017). Despite great efforts and numerous clinical trials, there is still a major need for effective therapies for SCM. Therefore, it is crucial to diagnose SCM early and intervene promptly to avoid the development of severe SCM. However, the diagnosis of SCM relies mainly on myocardial damage factors and echocardiography, which limits early intervention in SCM patients. Therefore, it is urgent to find new predictive biomarkers for the early diagnosis and intervention of SCM. In recent years, with the development of targeted gene therapy, targeted gene technology has achieved significant success in the early diagnosis, treatment, and prediction of prognosis in a variety of diseases (Bakhshinejad et al., 2014; Zeng et al., 2021), which provides a direction for early diagnosis of SCM. Ferroptosis is closely associated with the development of various cardiovascular diseases, including coronary artery disease, myocardial infarction and SCM (Fang et al., 2019; Wang et al., 2020). Recent studies have found that iron overload, an important pathological process of ferroptosis, is present in the heart tissue of septic mice (Sae-Khow et al., 2020), suggesting that ferroptosis may be a potential mechanism for the development of SCM. However, the impact of ferroptosis in the development of SCM is unclear. Therefore, we performed bioinformatics analysis based on SCM-related datasets to explore the role of ferroptosis in the pathogenesis of SCM.

With the development of public databases, microarray analysis is emerging as an effective method for identifying key molecules in various diseases, including septic cardiomyopathy (Ivandic et al., 2012). Therefore, in this study, we combined differentially expressed genes profiling in septic cardiomyopathy and FRGs analysis to construct PPI and miRNA-mRNA regulatory networks to improve our understanding of the molecular mechanisms of septic cardiomyopathy and to explore potential biomarkers.

After the construction of the PPI network, we identified 10 differentially expressed ferroptosis-related mRNA (Egfr, Ptgs2, Cdkn1a, Nfe2l2, Rela, Il6, Hmox1, Stat3, Myc and Vegfa) using cytoHubba. These hub genes may be potential targets for the regulation of ferroptosis in SCM. After validation, Cdkn1a, Ptgs2, Nfe2l2, Rela, and Vim were finally identified as key genes. These genes have been demonstrated to be involved in tumorigenesis, lipid peroxidation, inflammatory stress, or oxidative stress. Cdkn1a, an oncogene that encodes a potent cyclin-dependent kinase inhibitor, was reported to be specifically cleaved by CASP3-like caspases, which thus leaded to a dramatic activation of cyclin-dependent kinase2 and may be instrumental in the execution of apoptosis following caspase activation (Torgovnick et al., 2018). Moreover, Cdkn1a was upregulated in ferroptosis--related necrotizing enterocolitis (Bein et al., 2017) However, the role of Cdkn1a in SCM has not been investigated. In the present study, we further explored the effect of Cdkn1a on ferroptosis in SCM by western blotting analysis. The results show that the expression of Cdkn1a was up-regulated in HL-1 cells treated with LPS, but the expression of Gpx4 and FTH1, ferroptosis-related proteins, were down-regulated in LPS-treated HL-1 cells, however, Si-Cdkn1a treatment reversed this discrepancy, suggesting that Cdkn1a may be a potential regulatory target of LPS-induced ferroptosis in cardiomyocytes. Also, Ptgs2, also known as cyclooxygenase, was the marker gene of ferroptosis. Ptgs2 was reported to be significantly upregulated in LPS-induced septic cardiomyopathy mice (Li et al., 2020), which was consistent with our prediction, while PCR results further validated our prediction. Moreover, Nfe2l2 (Nuclear Factor, Erythroid 2 Like 2) is a Protein Coding gene. Li et al. reported that Curcumin alleviates LPS-induced oxidative stress, in bovine mammary epithelial cells via the Nfe2l2 signaling pathway (Li et al., 2021). However, the role of Nfe2l2 in LPS-induced myocardial injury has not been studied. These were consistent with our predicted results, and our PCR results further confirmed this. In addition, we found that the relationship between some mRNAs and sepsis is still poorly studied. Therefore, these key mRNAs are expected to be new biomarkers and therapeutic targets in future sepsis research.

Currently, many researchers have demonstrated that ferroptosis was associated with sepsis (Wei et al., 2020; Wang et al., 2021; Xiao et al., 2021). However, it is not common to construct miRNA-mRNA regulatory networks of FRGs in SCM and to predict its biomarkers. Here, we constructed a miRNA- FRGs network to explore the potential regulation of FRG by miRNAs in SCM. And 3 miRNA-miRNA regulatory pairs (mmu-miR-21-3p, mmu-miR-155-5p and mmu-miR-1892) were identified by bioinformatic analysis. And most of these miRNAs have been studied. Recent research implied that mmu-miR-21-3p was involved in oxidative stress during carcinogenesis (Kushwaha et al., 2020), while Zhou et al. found that miR-21-3p mediated angiogenesis in inflammatory states (Zhou et al., 2019). In addition, Zhang et al. reported that mmu-miR-155 promoted mastitis induced by *staphylococcus aureus* in mice (Zhang et al., 2019b). Finally, mmu-miR-1892-Cdkn1a was identified as key regulatory pair via validation. The PCR results showed that mmu-miR-1892 was downregulated in the LPS-induced myocardial injury model, which was consistent with our prediction. In addition, western blotting analysis results showed that Cdkn1a was up-regulated in the LPS-induced myocardial injury model, and interestingly, inhibition of its expression attenuated myocardial injury, suggesting that Cdkn1a may be a potential regulatory target in SCM. However, the specific mechanism of mmu-miR-1892-Cdkn1a in SCM has not been reported yet, and further studies are needed.

Sepsis is life-threatening organ dysfunction caused by a dysregulated host response to infection. Early identification and appropriate management in the initial hours after sepsis develops improves outcomes. Therefore, it is critical to select the most appropriate candidate drug intervention. At present, the main therapeutic drugs for sepsis are antimicrobials, norepinephrine, and hydrocortisone (Rhodes et al., 20162017). In this study, we used an online website to predict potential therapeutic agents associated with key genes and which may provide a reference for the pharmacological treatment of SCM.

## Conclusion

In the present study, we identified differentially expressed genes in SCM and analyzed the differential expression of FRGs in SCM by bioinformatics analysis. We constructed a miRNA-mRNA regulatory network in SCM and further obtained key FRGs and key miRNA-mRNA regulatory pair that may be potential regulatory mechanisms of myocardial ferroptosis in SCM. In addition, qRT-PCR and western blotting analysis were performed to confirm the abnormal expression of hub genes. The current study will contribute to the discovery of ferroptosis-related biomarkers in SCM and provide an important reference for further studies.

## Data Availability

The original contributions presented in the study are included in the article/Supplementary Material, further inquiries can be directed to the corresponding authors.
