# Supplementary figures and images for: Identification and Validation of Ferroptosis-Related Biomarkers in Septic Cardiomyopathy via Bioinformatics Analysis
Source: Front Genet. 2022 Apr 13;13:827559. doi: 10.3389/fgene.2022.827559 (PMC9043284; doi:10.3389/fgene.2022.827559)

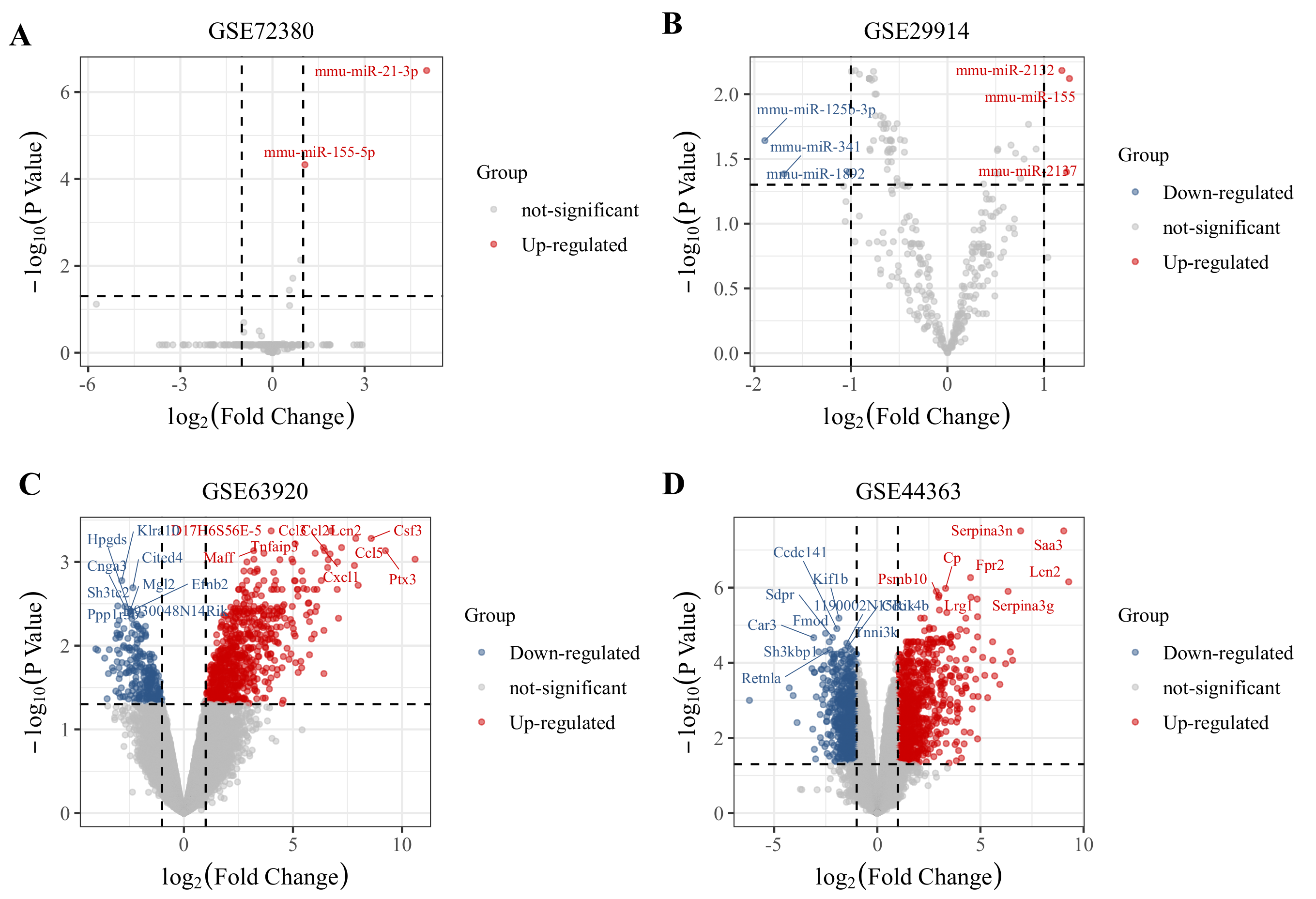

Supplement: Supplementary file 3 [file Image2.TIF]
